# Population pharmacokinetics of mefloquine, administered as a fixed-dose combination of artesunate-mefloquine in Indian patients for the treatment of acute uncomplicated *Plasmodium falciparum* malaria

**DOI:** 10.1186/1475-2875-13-187

**Published:** 2014-05-23

**Authors:** Vincent Jullien, Neena Valecha, Bina Srivastava, Bhawna Sharma, Jean-René Kiechel

**Affiliations:** 1 INSERM U1129, University Paris Descartes, Paris, France; 2 Service de Pharmacologie, Hôpital Européen Georges Pompidou; Assistance Publique - Hôpitaux de Paris, Paris, France; 3 National Institute of Malaria Research, New Delhi, India; 4 Drugs for Neglected Diseases initiative, F-79 Green Park Main, New Delhi 110016, India; 5 Drugs for Neglected Diseases initiative, 15 chemin Louis-Dunant, 1202 Geneva, Switzerland

**Keywords:** Mefloquine, Population pharmacokinetics, Adults, Malaria

## Abstract

**Background:**

Fixed-dose combinations of artemisinin combination therapy are strongly recommended to facilitate drug administration and compliance. New fixed-dose combinations must nevertheless be evaluated in relevant populations in terms of efficacy and pharmacokinetics.

**Methods:**

A single-arm, open-label, clinical trial was performed in Indian patients with acute uncomplicated *Plasmodium falciparum* malaria to investigate the efficacy and the pharmacokinetics of mefloquine when combined with artesunate in a fixed-dose combination (400/200 mg of mefloquine base/artesunate). The pharmacokinetic analysis was performed using a population approach.

**Results:**

Seventy-seven patients were included in the study. Mefloquine pharmacokinetics obeys a two-compartment model with first-order absorption and elimination. Mean parameter estimates (% inter-individual variability) were as follows: 0.16 h^-1^ (75%) for the absorption rate constant, 1.13 L/h (30%) for the apparent plasma clearance, 271 L (21%) for the apparent central distribution volume, 344 L (54%) for the apparent peripheral distribution volume, and 1.43 L/h for the apparent distribution clearance. These values were consistent with the pharmacokinetic results described in Thai patients. No significant covariate was found for clearance. Body weight explained the inter-individual variability of the apparent central and peripheral distribution volumes. The PCR-adjusted efficacy of the treatment was 100%.

**Conclusions:**

The lack of significant covariate explaining the inter-individual variability of mefloquine clearance, combined with the excellent efficacy, supports the use of the standard 200/400 mg of artesunate-mefloquine fixed-dose combination in Indian patients with uncomplicated *P. falciparum* malaria.

**Trial Registration:**

Clinical Trial Registration: ISRCTN70618692

## Background

Artemisinin combination therapy (ACT) is now the treatment of choice for uncomplicated *Plasmodium falciparum* malaria [1,2]. These combinations involve a rapidly eliminated and fast-acting artemisinin derivative, responsible for a rapid decline in the parasite biomass, together with a much slower eliminated drug that kills the remaining parasites. Mefloquine (MQ), is one of the partner drugs that can be combined with an artemisinin derivative, with the artesunate (AS)/MQ combination shown to be effective in several clinical trials [3-5], and used extensively in countries across Southeast Asia, the Western Pacific, Africa and Latin America over the last two decades [6]. The World Health Organization (WHO) currently recommends five formulations of ACT for the treatment of uncomplicated *P. falciparum* malaria, including the AS and MQ combination [7].

Fixed-dose combinations (FDC) are now strongly recommended compared to loose tablets because fewer tablets are involved and patient adherence should be improved [8]. The FDC of AS/MQ has been demonstrated to be efficacious and safe for uncomplicated malaria treatment in studies carried out in Thailand [9], Myanmar [10], India and Cambodia [11], as well as in a large intervention study on 23,845 patients in Brazil [12]. It is nevertheless necessary to verify not only the efficacy but also the pharmacokinetics of compounds administered as a FDC in a target population, in order to investigate possible population-related differences due to metabolism or food-drug interactions.

The present study investigated the efficacy, tolerability and pharmacokinetics of the AS/MQ FDC in Indian adult patients with acute uncomplicated *P. falciparum* malaria in highly endemic areas. Despite previous reports of high degrees of resistance to chloroquine in five Indian states, no MQ resistance was found [13]. The efficacy and safety results have already been reported [14], and here the population pharmacokinetics of mefloquine are presented.

## Methods

An open-label, single arm, multicentre study was carried out from December 2007 to November 2008 in patients of more than 18 years of age with *P. falciparum* malaria having an asexual parasitaemia density of between 1,000 and 100,000 parasites/μl of blood together with a fever ≥37.5°C. Patients with signs of severe malaria, febrile conditions due to a disease other than malaria, a history of anti-malarial treatment in the previous 15 days, anaemia, hepatic or renal impairment, a history of cardiovascular, respiratory, gastrointestinal, neurological, malignancy, psychiatric, or endocrine disorders were excluded. Pregnant or lactating patients were also excluded. The study was conducted in accordance with the local laws and regulations, Indian Good Clinical Practices, Ethical guidelines on biomedical research issued by the Indian Council of Medical Research and the International Conference on Harmonisation-Good Clinical Practices. The protocol was reviewed and approved by the Ethics Committee of the National Institute of Malaria Research, New Delhi, Goa Medical College and Hospitals, Goa and Kasturba Medical College, Mangalore. Written consent from each of the subjects were obtained before enrolment in the study.

Subjects were orally administered two tablets of AS/MQ FDC, containing 100 mg of AS and 200 mg of MQ base per tablet, once daily and over three consecutive days. Blood samples for analysis of MQ plasma concentrations were collected before dosing on D0, D3 (72 hours after first dosing), D7, and on one other occasion at a randomly selected time on day 28, 35 or 42.

An automated liquid chromatography – tandem mass spectrometry method ( LC-MS/MS) for the quantification of AS, MQ and dihydroartemisinin (DHA) levels in human plasma samples was developed and validated. These compounds were extracted from human plasma using a solid phase extraction procedure and injected into the liquid chromatograph coupled to a tandem mass spectrometric detector, and quantified by use of the internal standard method.

MQ was quantified using the Multiple Reaction Monitoring (MRM) transitions of 379.20➔ 361.atomic mass units (amu) and amodiaquine as an internal standard was quantified using MRM transitions of 356.70➔283.20 amu. A weighted linear regression using weighting 1/concentration^2^ was prepared to determine the concentrations of AS, MQ and DHA in human plasma. Eight-point calibration curves were prepared (20.2 ng/mL to 1,514.2 ng/mL for AS, 9.9 ng/mL to 3,455.5 ng/mL for MQ, and 39.2 ng/mL to 2,940.0 ng/mL for DHA), and used to determine concentrations of AS, MQ and DHA in subject samples. The Lower Limit of Quantification for MQ (LLOQ) was 9.9 ng/ml.

The bio-analytical method was validated for various validation parameters, such as specificity and selectivity, sensitivity, carry-over, solution linearity, precision and accuracy, recovery, stability, dilution integrity, matrix effect, re-injection reproducibility, and ruggedness.

During before study method validation for MQ the within batch precision (% CV) ranged from 2.14% to 6.99% and the between-batch precision ranged from 3.83% to 0.83%. The within batch accuracy (% Nominal) ranged from 90.91% to 114.41% and the between-batch accuracy ranged from 99.32 to 104.04%.

The between-run precision (%CV) for the Quality Control Samples of MQ during the study was 6.49%, 4.16% and 9.11% for LQC, MQC and HQC samples. The between-run accuracy (% Nominal) for the quality Control Samples of MQ during the study was 98.15%, 96.97% and 98.87% for the same samples respectively.

Concentration-time data were analysed by use of the first-order conditional estimation method with interaction of the non-linear mixed effects modelling program NONMEM (version VI, version 2.0, double precision) [13,15]. Three structural PK models (one, two, and three compartment models), with first-order absorption and first-order elimination from the central compartment were investigated. The estimated pharmacokinetic parameters were the absorption rate constant (ka), the apparent elimination clearance (CL/F, F being the bioavailability), the apparent central distribution volume (Vc/F) and, if relevant, apparent peripheral distribution volumes (Vp/F for a 2-compartment model, Vp_1_/F and Vp_2_F for a 3-compartment model) and distribution clearances (Q/F for a 2-compartment model, Q_1_/F and Q_2_/F for a 3-compartment model). Three different residual errors models (additive, proportional, and combined additive-proportionnal) were tested to investigate the residual variability. Interindividual variability was described by an exponential model, assuming that individual parameters arise from a multivariate lognormal distribution with mean vector and variance-covariance matrix to be estimated. The % interindividual variability was therefore calculated as the root mean square of the omega^2^ value given by NONMEM. Systematic testing for the influence of continuous covariates on the pharmacokinetic parameters was done by use of a generalized allometric model, according to the following equation, by using, for example, *CL/F* and *BW*:

CLF=TVCLF×BWmedianBWθ

where *TV(CL/F)* was the typical value of the apparent clearance for a patient with the median covariate value, and *θ* was the influential factor for body weight (BW).

Continuous tested covariates for clearance were age, bodyweight, parasitaemia, hepatic enzymes, haemoglobin, and serum creatinin. Continuous covariates tested for distribution volume were bodyweight, haemoglobin, and parasitaemia.

The possible influence of the day of administration on CL/F was investigated as follows:

CLF=TV1CLF×OCC+TV2CLF×1-OCC

Where TV_1_(CL/F) is the mean apparent clearance for the first two days of treatment and TV_2_(CL/F) is the mean apparent clearance for the third day of treatment. OCC(occasion) is equal to 1 for the first two days of treatment, and otherwise to 0.

The significance of a relationship between a pharmacokinetic parameter and a covariate was assessed by use of the Chi-square test of the difference between the objective functions of the basic model (without the covariate) and the model with the covariate. A covariate was retained in the model if it produced a minimum decrease in the objective function of 3.64 units (*P* =0.05, 1 degree of freedom) and if its effect was biologically plausible. An intermediate multivariate model that included all selected covariates was then obtained. A covariate was retained in the final multivariate model if its deletion from the intermediate model led to a 6.63-point increase in the objective function (*P* =0.01, 1 degree of freedom). At each step, the goodness of fit was evaluated by use of a graph of the weighted residuals *versus* time after administration of the dose (time) or *versus* the predicted concentrations. Normalized predictions errors (NPDE) *versus* time and predicted concentrations were used to assess the lack of bias of the final model. NPDE are computed as the quantiles of the observations in the predicted distribution, obtained for each observation by simulating 1000 datasets using the model and the design of the original dataset. The computation also involves a decorrelation step to account for the correlation induced by the multiple observations within one subject. The distribution of the NPDE under the assumption that the model describes appropriately the observed data is the standard Gaussian distribution, and graphs of NDPE versus time and versus predicted concentrations can be used to evaluate this assumption [16].

The accuracy and robustness of the final population models were assessed by a visual predictive check. The final population model parameters were used to perform 1000 simulations of the database. The 5^th^ and the 95^th^ percentiles as well as the 50^th^ (median) of simulated concentrations were plotted against observed concentrations.

Bayesian estimates of the individual pharmacokinetic parameters (obtained in NONMEM output) were used to calculate individual MQ elimination half-life, the individual area under the curve from time zero to infinite (AUC), and MQ concentration at D7 (C7) and D28 (C28). Elimination half-life was calculated as follows: ln2/β with β = 0.5 × (Q/Vc + Q/Vp + CL/Vc-[(Q/Vc + Q/Vp + CL/Vc)^2^ – 4 × Q/Vp x CL/Vc]^1/2^) and the calculation of C7 and C28 was made by NONMEM.

The primary efficacy outcome of the study was the PCR-uncorrected cure rate (proportion of patients with an adequate clinical and parasitological response) on D63 and PCR-corrected cure rate on D63 (proportion of patients without recrudescence/ inconclusive/ no results as classified by PCR genotyping).

Comparison of MQ C7 and C28 between treatment success and treatment failure was expected to be performed by non-parametric statistical analyses.

## Results

**Figure 1:**
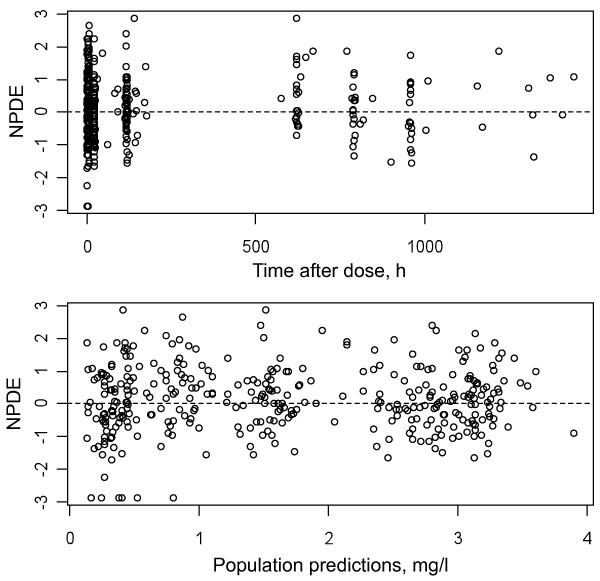
**Lack of bias evidenced for the final model by the normalized predictions errors (NPDE) *versus *** **time after dose (T1), expressed in hours, and population predictions (PRED).**

Seventy-seven patients (74 men, three women) were included. Their demographic and biological characteristics are given in Table 1. Three-hundred and fifty-five concentrations of MQ were available for the analysis. Distribution of sampling times can be seen on Figure 1.

**Table 1:** Characteristics of the study population at the first drug intake

	**Mean**	**Standard deviation**	**Range**
Age (y)	28.1	9.0	18-45
Body weight (kg)	53.3	7.3	40-70
Parasite density (count/μL)	8356*	NR	1165-94693
Haemoglobin (mg/dL)	13.1	2.15	7.1-16.8
ASAT (IU)	34.4	14.1	18-77
ALAT (IU)	26.2	17.1	1-85
Serum creatinine	0.96	0.18	0.6-1.7

*Median; *NR*: not relevant.

*ASAT* aspartate aminotransferase; *ALAT* alanine aminotransferase.

(n = 77: 74 male/3 female).

The optimal model was a two-compartment model with first-order absorption and elimination. The estimated parameters were the absorption rate constant (ka), the apparent central distribution volume (Vc/F), the apparent peripheral distribution volume (Vp/F), the apparent elimination clearance (CL/F), and the apparent distribution clearance (Q/F), where F is the bioavailability. Inter-individual variability of Q/F could not be estimated. Residual variability was described by a proportional error model.

BW was the only significant covariate and was found to explain the inter-individual variability of Vp/F and Vc/F. All other covariates, including the day of dosing was not found to significantly influence the inter-individual variability of the PK parameters. The parameter estimates are given in Table 2.

**Table 2:** Parameter estimates

	**Base model**		**Final model**	
**Parameter**	**Estimate**	**SD**	**Estimate**	**SD**
Ka (h^-1^)	0.166	0.0266	0.163	0.0263
CL/F (L/h)	1.15	0.0496	1.13	0.0501
Vc/F (L)	279	14.7	271	14.1
Ѳ_BW,Vc_	/	/	0.87	0.288
Q/F (L/h)	1.33	0.124	1.43	0.152
Vp/F (L)	341	39	344	40.6
Ѳ_BW,Vp_	/	/	2.41	1.08
$\omega_{\mathrm{ka}}^{2}$	0.675	0.250	0.562	0.232
ωCLF2	0.0939	0.0224	0.0894	0.0218
ωVcF2	0.0603	0.0209	0.0453	0.0163
ωVpF2	0.401	0.166	0.295	0.128
σ^2^	0.0689	0.0150	0.0692	0.015

*SD* standard deviation of the estimate (obtained from the covariance step); Ka: absorption rate constant; CL/F: apparent oral clearance; Vc/F: apparent central distribution volume; Ѳ_BW,Vc_: influential factor of BW on Vc/F; Q/F: apparent distribution clearance; Vp/F: apparent distribution volume ; Ѳ_BW,Vp_ : influential factor of BW on Vp/F ; ωCLF2: inter-individual variability of CL/F; ωVcF2: inter-individual variability of Vc/F; ωVpF2: inter-individual variability of Vp/F; $\omega_{\mathrm{ka}}^{2}$: inter-individual variability of Ka; σ^2^: proportional residual error.

**Figure 2:**
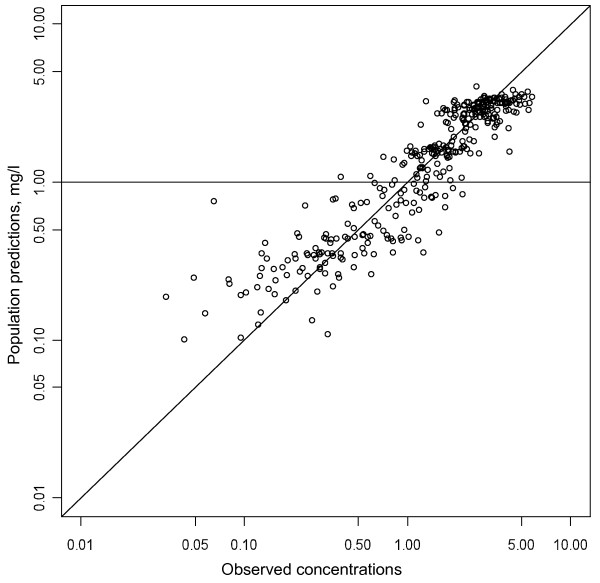
**Population predictions versus observed mefloquine concentrations.** Solid diagonal line: identity (y = x) line.

**Figure 3:**
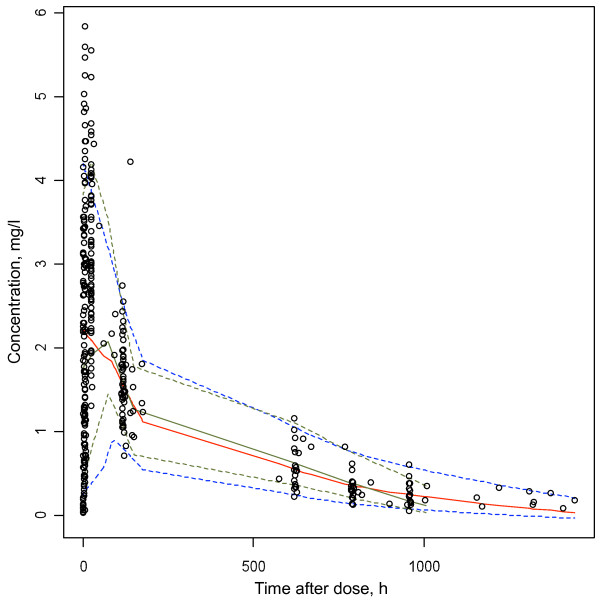
**Visual predictive checks for the final model.** Open circles: observed concentrations; T1: Time post-dose (hours); red solid line: 50^th^ percentile of the simulated concentrations; upper blue dotted line: 95^th^ percentile of the simulated concentrations; lower blue dotted line: 5^th^ percentile of the simulated concentrations, solid green line: 50^th^ percentile of observed concentrations, lower and upper dotted green lines: 5^th^ and 95^th^ percentiles of the observed concentrations respectively.

Epsilon shrinkage was 19.8%, eta shrinkage was 21% for CL/F, 34% for Vc/F, 53% for Vp/F, and 20% for ka.The lack of bias of the final model is observed in Figures 1 and 2. Visual predictive checks for the final model are displayed in Figure 3 and 11.4% of the observed concentrations were outside the 90% confidence interval.

Median (range) mefloquine AUC was 1055 (596 – 1741 mg.h/l). Median (range) C7 was 1.58 (1.08 – 2.90) mg/l, and median (range) C28 was 0.29 (0.11 – 0.57) mg/l. Since no recrudescence and only one new infection was observed over the follow-up of the study (PCR-corrected cure rate of 100%), no correlation between MQ C7 or C28 and treatment efficacy could be investigated.

## Discussion

The present model accurately described the data and was in agreement with previous results seen in adult patients with uncomplicated malaria (Table 3), even though the present study was performed on plasma samples and most of these previous results were based on the concentration of MQ in whole blood. This can be explained by the blood to plasma ratio of MQ which is close to 1 [17]. This consistency with results from studies performed in non-Indian patients suggests there are no ethnic-related differences in MQ pharmacokinetics, so a similar dosing regimen should achieve similar concentrations in Thai and in Indian patients. This result also confirms the bioavailability of the dosage form used in the present study (a FDC of AS and MQ) is similar to the bioavailability of the non-fixed dose MQ forms that were used in previous studies described by Krudsood *et al.*[18]. A slightly longer elimination half-life was found in the present study, which may be due to differences in study design and in the sensitivity of the analytical methods. Indeed, delayed sampling times were available in the present study, and the quantification of MQ was possible in these samples thanks to the low LLOQ of the analytical method (9.9 ng/ml, compared to previous LLOQs of about 50 ng/ml (Krudsood, Simpson), 70 ng/ml (Ashley), 100 ng/ml (Charles), which allowed the determination of the two-compartment disposition model.

**Table 3:** Pharmacokinetics parameters of mefloquine in the present work and in previous studies

**Parameter**	**Gutman** ***et al*.**[19]	**Charles** ***et al*.**[20]	**Ashley** ***et al.*** [21]	**Krudsood** ***et al*.**[18]	**Current study**
Country of study	Peru	Australia	Thailand	Thai-Burmese border	India
context	uncomplicated falciparum malaria	prophylaxis	uncomplicated falciparum malaria	uncomplicated falciparum malaria	uncomplicated falciparum malaria
Age (yr): Mean (range)					
	36	26	19 (Median)	27.8	28
	18 - 61	18 - 55	2 - 55	16 - 50	18 -45
BW (kg)					
Mean	NA	82	44.5 (Median)	51	53.3
Range	NA	53 -135	10 - 63	40 - 65	40 - 70
CL/F	0.017 L/h/kg	2.09 L/h	1.33 L/h	0.024 L/h/kg	1.13 L/h
					0.022 L/h/kg
V/F	8.57 L/kg	1011 L	488 L	NA	615 L
					11.9 L/kg
T_1/2_	14.5 days	14 days	10.5 days	13.4 days	21.6 days

*NA* not available.

Differences in study design probably also explain why no modification in MQ pharmacokinetics during the treatment administration period could be detected. This change was previously described and attributed to the rapid clinical improvement seen due to treatment efficacy [22]. There was indeed only one sample drawn within the first three days of treatment, which precluded the identification of early changes in MQ pharmacokinetics.

**Figure 4:**
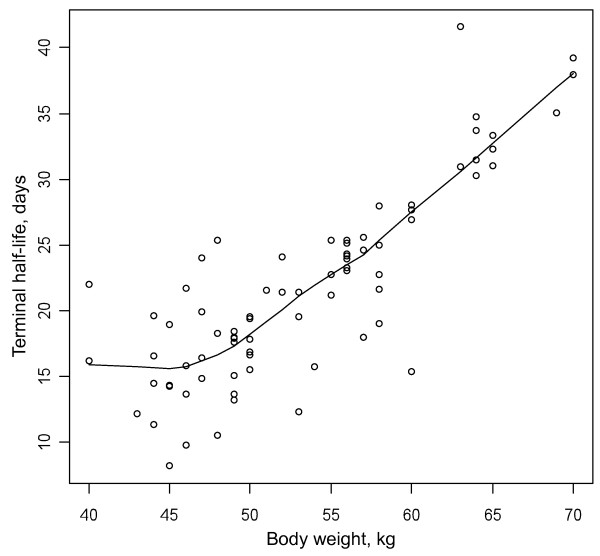
Relationship between mefloquine terminal half-life (days) and body weight (kg).

In agreement with previous findings [20], BW did not account for the inter-individual variability of MQ CL/F, so lending support for the current use of a constant dose, independent of BW, from one adult subject to another, consistent with previous population pharmacokinetics model performed in adults [20]. These results contrast with those of Ashley *et al*. [21], who reported increasing BW was associated with a reduction in MQ clearance, however this study included both children and adults so the relationship between clearance and BW may have been contributed by the paediatric population. Nevertheless, a significant relationship between BW and the central and peripheral distribution volumes was found, with a proportional increase in MQ elimination half-life with increasing BW observed (Figure 4). The prolonged presence of MQ in subjects with higher BW may have some impact on the prevention of re-infection, since it will take more time for MQ levels to fall below effective concentration levels [23]. However, the practical consequences of this theoretical finding should be further investigated. A similar increase in the clearance and distribution volume of MQ, secondary to combination with artesunate, and leading to a decrease in MQ Cmax and AUC of about 25%, was previously reported by Karbwang *et al.*[24]. The mechanism of this interaction could rely on a decrease in the bioavailability or in the protein binding of MQ. To our knowledge, this PK interaction was not further investigated. Anyway, because of the good efficacy of the AS/MQ combination, the clinical relevance of this interaction is unclear.

## Conclusions

The present study provided population pharmacokinetics parameters for MQ, administered as a FDC of AS/MQ, in Indian adult patients with acute uncomplicated *P. falciparum* malaria. The lack of relevant biological covariates on MQ CL/F, combined with the excellent efficacy results that were obtained, support the use of the 200/400 mg dose of AS/MQ in this population.

## Abbreviations

ACT: Artemisinin combination therapy; AS: Artesunate; ASAT: Aspartate aminotransferase; ALAT: Alanine aminotransferase; BW: Body weight; DHA: Dihydroartemisinin; MQ: Mefloquine; NPDE: Normalized predictions errors; PRED: Population predictions.

## Competing interests

The authors have declared that they have no competing interests.

## Authors’ contributions

VJ pharmacokinetic analysis, writing of the manuscript; BSh study coordination and monitoring; NV study management and execution; BSr technical overview; J-RK study pharmacokinetics protocol, contribution to the manuscript and discussion of the results. All authors read and approved the final manuscript.
